# Human Development Index (HDI) of the maternal country of origin as a predictor of perinatal outcomes - a longitudinal study conducted in Spain

**DOI:** 10.1186/s12884-017-1515-1

**Published:** 2017-09-21

**Authors:** S. Garcia-Tizon Larroca, J. Arevalo-Serrano, A. Duran Vila, M. P. Pintado Recarte, I. Cueto Hernandez, A. Solis Pierna, S. Lizarraga Bonelli, J. De Leon-Luis

**Affiliations:** 1 Department of Obstetrics and Gynaecology, Hospital General Universitario Gregorio Marañón, Universidad Complutense de Madrid, 28029 Madrid, ES Spain; 2 Department of Internal Medicine, Hospital Universitario, Principe de Asturias de Alcalá de Henares, Madrid, Spain

**Keywords:** Human development index, Perinatal health, Perinatal mortality, Maternal mortality, Low Birth weight, Preterm delivery, Immigrants, Socioeconomic status

## Abstract

**Background:**

In an era of worldwide population displacement, recent studies have identified strong associations between social situations and perinatal outcomes among immigrants. Little is known about the effect of maternal social background on pregnancy outcomes. The Human Development Index (HDI) assesses the following dimensions of human development: life expectancy, education level and income. The objective of our study was to determine if maternal HDI may be used to identify women at increased odds of poor pregnancy outcomes.

**Methods:**

We conducted a longitudinal population-based study in a tertiary centre in Madrid, Spain. The outcome variables were maternal and perinatal/antenatal mortality, preeclampsia (PE), low birth weight (LBW), gestational diabetes mellitus (GDM), preterm delivery (PTD) before 37 and 34 gestational weeks, abnormal cardiotocography (CTG) during delivery, C-section (CS) due to abnormal CTG, pH < 7.10 at birth, Apgar at 5 min ≤ 7, and resuscitation type ≥3. We performed multivariate logistic regression analyses adjusted for potential confounding variables to evaluate the associations between maternal HDI and perinatal outcomes.

**Results:**

In total, 38,719 singleton infants who were born in our maternity ward between 2010 and 2016 and had perinatal outcome data available were included in this study. The neonates of women from medium/low HDI countries had significantly lower odds of low birth weight (LBW) than their very high HDI country counterparts (OR 0.63, 95% CI 0.55–0.72). However, the odds of PTD before 37 gestational weeks and PE were higher in the medium/low HDI group than the very high HDI group (OR 1.26, 95% CI 1.04–1.53; OR 1.35, 95% CI 1.02–1.79, respectively). Poorer neonatal outcomes were identified in the medium/low HDI group than the very high HDI group, including greater odds of abnormal CTG, CS due to abnormal CTG and Apgar 2 ≤ 7 (*p* < 0.05).

**Conclusions:**

Our findings suggest that the infants of mothers from medium/low HDI had lower odds of LBW but higher odds of PTD, PE and poor neonatal outcomes. These results support the hypothesis that maternal HDI can be used to understand the impact of maternal origin on pregnancy outcomes. Further studies are needed to confirm its validity.

## Background

Social determinants comprise a broad range of factors associated with health and access to care, including income inequality, social exclusion, sense of collective efficacy and social connectedness. Pregnancy has also been found to be strongly influenced by social situations. Further, in pregnancy, social deprivation has been found to be associated with poorer gestational outcomes such as preterm birth, infant mortality and growth retardation [1–3].

Certain population groups may be more likely than others to suffer from these and other health risks because of geographical, economical and educational factors [4]. Previous studies have utilized a variety of measures to assess social situations, including educational level, other socioeconomic indicators and complex scores [5].

Regarding immigrants pregnancy care perinatal and neonatal health outcomes have sometimes been found to be worse and other times been found to be better in home than host countries.

The results of previous studies on perinatal outcomes in immigrants have been heterogeneous, as their measures of derivation were dependent upon the adjustment for variables that often differ widely across the study groups; additionally, the study designs and manner in which of socioeconomic factors were integrated differed between studies. Maternal socioeconomic status in this population has been assessed in many different ways by race/ethnicity, foreign-born status, education or deprivation [6]. This vast existing body of scientific evidence has revealed the presence of an “immigrant paradox,” which suggests that women of lower socioeconomic status may have better pregnancy outcomes than native women [7].

To more accurately quantify social vulnerability and its multifactorial aspects, some groups have developed different indexes that have rarely been suitable for the evaluation of pregnancy outcomes. To identify women at increased odds of poor birth outcomes, indicators of deprivation should incorporate relevant multidimensional measures.

The Human Development Index (HDI) is a summary measure of a country’s average level of achievement in the following major dimensions of human development: living a long and healthy life, being knowledgeable and having a decent standard of living. Life expectancy serves as an indicator of the health dimension; standard of living is measured in terms of gross national income per capita (GNI) and education level is evaluated as the average number of years of schooling among adults aged 25 years and older and expected of number of years of schooling among children [8].

A country obtains a higher HDI score when its population has a higher life expectancy, education level, and GNI per capita; these scores are reported within the annual Human Development Report published by the United Nations Development Programme (UNDP) [9].

Life expectancy data were provided by the UN Population Division; data on the mean number of years of schooling were obtained from the UNESCO Institute of Statistics; and GNI per capita data were provided by the World Bank and International Monetary Fund.

Maternal HDI might be a useful manner in which to identify women at increased odds of poor perinatal outcomes, as it is designed to reflect basic aspects of human development that are also relevant to prenatal care.

## Methods

We conducted a longitudinal cohort study including women who delivered in the Maternidad de O’Donnell de Madrid, Spain (Hospital General Universitario Gregorio Marañon de Madrid). Data on all singleton births that occurred in our centre during the period between January 2010 and December 2016 were obtained.

Data on maternal and pregnancy characteristics were collected during prenatal visits or at the time of admission to the hospital ward. Participants completed a questionnaire that collected information on their date and place of birth, history of pregestational diabetes and parity. All information provided was reviewed with the women by a doctor or midwife.

The HDI for each mother’s country of origin was identified based on the UNDP human development report published for the year of delivery. The HDI index is the geometrical mean of the three dimension indices:

$$ \mathrm{HDI}={\left({\mathrm{I}}_{\mathrm{Health}}.{\mathrm{I}}_{\mathrm{Education}}.{\mathrm{I}}_{\mathrm{I}\mathrm{ncome}}\right)}^{1/3} $$

Maximum and minimum values are set in order to transform the indicators expressed in different units into indices on a scale of 0 to 1. These goalposts act as the “natural zeros” and “aspirational targets” from which component are standardized (Table 1).

**Table 1:** HDI and its components

Dimension	Indicator	Minimum	Maximum
Health	Life expectancy (years)	20	85
Education	Expected years of schooling (years)	0	18
	Mean years of schooling (years)	0	15
Standard of living	Gross national income per capita (2011 PPS$)	100	75,000

These are examples of countries listed by HDI as included in the UNDP human development report in 2016:

- Very High HDI (> 0.80): Norway (0.949), Germany (0.926), Spain (0.884), Croatia (0.827).
- High HDI (0.70–0.80): Bulgaria (0.794), Cuba (0.775), China (0.738), Uzbekistan (0.701).
- Medium HDI (0.55–0.70): Moldova (0.699), Guatemala (0.640), India (0.624), Zambia (0.579).
- Low HDI (< 0.55): Syria (0.536), Afghanistan (0.479), Burundi (0.404), Niger (0.353).

Countries fall into four HDI categories. The first two groups are referred as *developed countries* and the last two are considered *developing countries*.

In order to simplify the analysis we created a group called “Medium-Low HDI” that contained the last two categories so we could differentiate patients that belonged to Very High, High and Medium-Low HDI of the maternal country of origin.

The outcome measures were perinatal/antenatal mortality, preeclampsia (PE), low birth weight (LBW), maternal mortality, gestational diabetes mellitus (GDM), preterm delivery (PTD) before 37 gestational weeks, PTD before 34 gestational weeks, abnormal cardiotocography (CTG) during delivery, C-section (CS) due to abnormal CTG, pH at birth < 7.10, Apgar at 5 min ≤ 7, and resuscitation type ≥ 3.

We excluded women with multiple pregnancies, miscarriages and foetal deaths before 22 weeks of gestation.

PE was diagnosed based on the guidelines of the International Society for the Study of Hypertension in Pregnancy; these guidelines define PE as the measurement of a systolic blood pressure of 140 mmHg or greater and/or a diastolic blood pressure of 90 mmHg or greater on at least two occasions 4 h apart after 20 weeks of gestation. Proteinuria was defined as a urinary albumin-creatinine ratio greater than 300 mg in a 24 h urine collection or two dipstick readings of ++ or higher.

GDM was diagnosed using a two-step approach. The O’Sullivan’s test was performed on all women between 24 and 26 gestational weeks; this screening test involves measuring glucose levels one hour after the administration of 50 g oral glucose. The test results are considered positive when the glucose value is 140 mg/dl or higher. A 100 g oral glucose tolerance test was performed on those who had positive screening test results to establish the diagnosis of GDM.

PTD before 34 and 37 completed gestational weeks included pregnancies with spontaneous onset of labour with or without preterm rupture of membranes; however, those with iatrogenic delivery were excluded.

LBW neonates were defined as those with a birth weight below the 10th percentile for gestational age.

Based on standards set by the World Health Organization (WHO) and the National Centre for Health Statistics (NCHS) of the Centers for Disease Control and Prevention (CDC), perinatal mortality and foetal mortality were defined as deaths occurring less than 7 days after birth and at a gestational age of 28 weeks or older, respectively [10].

The present study was approved by the Ethical Committee of Hospital General Universitario Gregorio Marañon de Madrid (Comité Ético de Investigacion Clínica, reference number OBS05042016).

### Statistical analysis

We performed multivariate logistic regression analyses to estimate odds ratios (ORs) for the associations between maternal HDI and perinatal outcomes after adjustment for maternal age, pregestational diabetes history and parity. Maternal HDI served as a predictor variable and was divided in three categories: very high, high and medium/low. The women were then divided into these three groups based on the HDI of their country of origin.

First, chi-square and Fisher’s exact tests were used to identify significant between-group differences in categorical variables. Crude associations between maternal HDI and perinatal outcomes were calculated, and two-by-two multiple comparisons were performed with *p*-values adjusted using Holm’s method.

The effect of maternal HDI on each perinatal outcome was explored using backward multivariate binary logistic regression.

Variable selection in the multivariate regression model was performed according to subject matter knowledge considered relevant predictors of perinatal outcome. Order of selection to evaluate the inclusion or exclusion of predictors was performed by descending statistical significance. Criteria to keep or retire predictors in the model took into account the clinical significance, that is, a variation of more or less than 10% of maternal HDI OR.

The odds ratios derived based on the results of the logistic regression and Wald test *p*-values were used to assess the statistical significance of the predictor variables within the models. The likelihood-ratio test was used to assess the fit of the model.

The significance level was set at α = 0.05, and all analyses were performed using SPSS 18 (SPSS Inc., Chicago, IL, USA) software.

## Results

In total, 38,719 singleton infants who were born during the study period and for whom perinatal outcome data available were included in this study. The variables with the most missing values were pH at birth (0.6%) and birth weight below the 10th percentile for gestational age (0.3%).

Seventy percent of infants were born to patients from very high HDI countries; women from high HDI countries represented a 23% of the sample, and 7.1% of infants had mothers who originated from medium/low HDI countries.

Information on the sample as parity, maternal age, pregestational diabetes (variables of adjustment in the model) for each HDI category are shown in Table 2. The following four maternal deaths occurred after delivery in our centre: two cases of postpartum haemorrhage refractory to medical and surgical interventions, 1 case of chorioamnionitis at 26 weeks in a patient diagnosed with metastatic melanoma and 1 case of idiopathic intracranial hypertension with intracerebral haemorrhage and pneumonia after mechanical ventilation.

**Table 2:** Maternal age, parity and pregestational Diabetes for each HDI category

	Group A:	Group B:	Group C:	*p*-value
	Very High HDI	High HDI	Medium-Low HDI	Global
Maternal Age (IQR)	33 (30–37)	31 (26–35)	31 (27–35)	<0.001
Parity (IQR)	1 (1–2)	1 (1–2)	2 (1–2)	<0.001
Pregestational Diabetes n/N (%)	132/26955 (0.49)	18/8862 (0.20)	7/2744 (0.25)	0.001

Comparisons between HDI groups and perinatal outcomes are presented in Table 3.

**Table 3:** Comparisons between HDI groups and perinatal outcomes

	Group A:	Group B:	Group C:	*p*-value
	Very High HDI	High HDI	Medium-Low HDI	Global
Perinatal/antenatal death	120 (0.44)	32 (0.36)	10 (0.36)	0.519
Maternal death	2 (0.01)	2 (0.02)	0 (0.00)	0.446
Preeclampsia	417 (1.5)	140 (1.6)	57 (2.1)	0.103
LBW	3862 (14.3)	844 (9.5)	261 (9.5)	<0.001
PTD < 37 w	959 (3.5)	386 (4.3)	122 (4.4)	<0.001
PTD < 34 w	332 (1.2)	128 (1.4)	37 (1.3)	0.280
Abnormal CTG	1247 (4.6)	467 (5.3)	180 (6.6)	<0.001
CS due to abnormal CTG	1104 (4.1)	414 (4.7)	161 (5.9)	<0.001
Ph < 7.10	587 (2.2)	218 (2.5)	66 (2.4)	0.244
Apgar 2 ≤ 7	306 (1.1)	119 (1.3)	45 (1.6)	0.033
Resuscitation > 3	1783 (6.6)	660 (7.5)	205 (7.5)	0.010
GDM	768 (2.8)	199 (2.2)	81 (2.9)	0.008

The initial crude associations and multiple comparisons between HDI groups and perinatal outcomes showed the following significant results, as seen in Table 4. In this analysis, we found that the rate of LBW was higher in the very high HDI group than the medium/low HDI group (OR 1.6, 95% confidence interval [CI] 1.4–1.8). We did not identify statistically significant between-group differences in the rates of PE, maternal mortality, neonatal-perinatal mortality or PTD before 34 weeks of gestation.

**Table 4:** Significant multiple comparisons between HDI groups and perinatal outcomes

	*p*	OR	CI 95%
LBW	<0.001		
Group A: Very High HDI – Group B: High HDI	<0.001	1.6	1.5–1.7
Group A: Very High HDI – Group C: Medium-Low HDI	<0.001	1.6	1.4–1.8
PTD < 37 w	<0.001		
Group A: Very High HDI – Group B: High HDI	0.001	0.8	0.7–0.9
Group A: Very High HDI – Group C: Medium-Low HDI	0.034	0.8	0.7–1.0
Abnormal CTG	<0.001		
Group A: Very High HDI – Group B: High HDI	0.020	0.9	0.8–1.0
Group A: Very High HDI – Group C: Medium-Low HDI	<0.001	0.7	0.6–0.8
Group B: High HDI – Group C: Medium-Low HDI	0.020	0.8	0.7–1.0
CS due to abnormal CTG	<0.001		
Group A: Very High HDI – Group B: High HDI	0.024	0.9	0.8–1.0
Group A: Very High HDI – Group C: Medium-Low HDI	<0.001	0.7	0.6–0.8
Group B: High HDI – Group C: Medium-Low HDI	0.024	0.8	0.7–1.0
Resuscitation > 3	0.010		
Group A: Very High HDI – Group B: High HDI	0.018	0.9	0.8–1.0
GDM	0.008		
Group A: Very High HDI – Group B: High HDI	0.009	1.3	1.1–1.5

After adjusting for maternal age, pregestational diabetes history and parity (Table 5), the results of our multivariate model indicated that the rate of LBW was lower in the medium/low HDI group than the very high HDI group (aOR 0.63, 95% CI 0.55–0.72). Additionally, the rate of PTD before 37 gestational weeks was higher in the medium/low HDI group than the very high HDI group (aOR 1.26, 95% CI 1.04–1.53) and PE was identified significantly more frequently in women in the medium/low HDI group than women in the very high HDI group (aOR 1.35, 95% CI 1.02–1.79). The proportion of women whose records indicated the occurrence of abnormal CTG during labour was higher in medium/low HDI group than the very high HDI group (aOR 1.45, 95% CI 1.23–1.70); accordingly, the rate of CS due to abnormal CTG was higher in the medium/low HDI group than very high HDI group (aOR 1.46, 95% CI 1.23–1.73). Apgar 2 scores ≤ 7 were more frequently identified in the medium/low HDI group than the very high HDI patients (aOR 1.45, 95% CI 1.06–1.99), and resuscitation type ≥ 3 was documented more frequently in the records of high HDI patients than those of very high HDI patients (aOR 1.14, 95% CI 1.04–1.25).

**Table 5:** Multivariate logistic regression adjusted for maternal age, pregestational diabetes and parity

	N	*p*	aOR	CI 95%
Perinatal/antenatal death	38,718	0.51		
Global Maternal HDI		0.52		
High HDI – Very High HDI		0.298	0.81	0.55–1.20
Medium-Low HDI – Very High HDI		0.547	0.82	0.43–1.57
Maternal death	38,718	0.412		
Global Maternal HDI		0.537		
High HDI – Very High HDI		0.265	3.05	0.43–21.66
Medium-Low HDI – Very High HDI		0.988	0	0.00 -
Preeclampsia	38,718	0.124		
Global Maternal HDI		0.105		
High HDI – Very High HDI		0.806	1.02	0.85–1.24
Medium-Low HDI – Very High HDI		0.034	1.35	1.02–1.79
LBW	38,535	<0.001		
Global Maternal HDI		<0.001		
High HDI – Very High HDI		<0.001	0.63	0.58–0.68
Medium-Low HDI – Very High HDI		<0.001	0.63	0.55–0.72
PTD < 37 weeks	38,718	0.001		
Global Maternal HDI		<0.001		
High HDI – Very High HDI		0.001	1.24	1.10–1.40
Medium-Low HDI – Very High HDI		0.017	1.26	1.04–1.53
PTD < 34 weeks	38,718	0.288		
Global Maternal HDI		0.281		
High HDI – Very High HDI		0.117	1.18	0.96–1.45
Medium-Low HDI – Very High HDI		0.59	1.1	0.78–1.55
Abnormal CTG	38,582	<0.001		
Global Maternal HDI		<0.001		
High HDI – Very High HDI		0.012	1.15	1.03–1.28
Medium-Low HDI – Very High HDI		<0.001	1.45	1.23–1.70
CS due to abnormal CTG	38,590	<0.001		
Global Maternal HDI		<0.001		
High HDI – Very High HDI		0.018	1.15	1.03–1.29
Medium-Low HDI – Very High HDI		<0.001	1.46	1.23–1.73
pH < 7.10	38,474	0.25		
Global Maternal HDI		0.245		
High HDI – Very High HDI		0.112	1.14	0.97–1.33
Medium-Low HDI – Very High HDI		0.87	1.11	0.86–1.44
Apgar 2 ≤ 7	38,590	0.04		
Global Maternal HDI		0.034		
High HDI – Very High HDI		0.113	1.19	0.96–1.47
Medium-Low HDI – Very High HDI		0.02	1.45	1.06–1.99
Resuscitation >3	38,590	0.011		
Global Maternal HDI		0.01		
High HDI – Very High HDI		0.006	1.14	1.04–1.25
Medium-Low HDI – Very High HDI		0.084	1.14	0.98–1.33
GDM	38,717	0.007		
Global Maternal HDI		0.008		
High HDI – Very High HDI		0.003	0.79	0.67–0.92
Medium-Low HDI – Very High HDI		0.743	0.8	0.82–1.31

Statistically significant between-group differences were identified for maternal mortality neonatal-perinatal mortality, PTD before 34 weeks of gestation or pH at birth < 7.10 in the final model.

## Discussion

To our knowledge, this is the first study to investigate maternal HDI as the main social determinant of perinatal and pregnancy health. However, a recent study utilized maternal origin to define HDI scores and evaluate the extent to which stillbirths affected international comparisons of preterm delivery rates in low income countries [11].

This HDI simplifies and captures major social characteristics and encompasses various aspects of human development across countries in the form of a common score. Therefore, maternal origin can be categorized not only by race and ethnicity but also by income and educational level.

The aim of this paper was to identify differences in pregnancy outcomes between groups of patients demonstrating strong underlying inequalities that might interfere with antenatal care.

Our results demonstrate that after adjusting for potential confounding variables, a significant association between maternal HDI and a range of pregnancy complications still remained.

In summary, we can infer that patients with medium/low HDI 1) did not have an excess risk of maternal or perinatal mortality; 2) had lower odds of LBW; 3) had greater odds of PTD before 37 weeks of gestation; 4) had greater odds of PE; and 4) had poorer neonatal outcome, including a higher rates of abnormal CTG and CS due to abnormal CTG and lower Apgar scores at 5 min.

A key finding of this study was that belonging to the lower HDI group was associated with lower odds of LBW when compared with women from MEDC. This finding confirms the results of previous studies wherein the risk of poor prenatal outcome was greater among native-born than immigrant mothers [12]. Several factors may have contributed to the better pregnancy outcomes identified in this group, and especially newborn weight. Immigrants tend to engage in healthier behaviours, such as consuming less alcohol and cigarettes and having healthier diets and stronger family ties. Another possible explanation for this finding is aligned with the “healthy migrant effect,” which suggests that a minimum level of health is required to migrate; thus, only a segment of the healthy population will migrate to the host country [13].

Another study evaluated areas with high levels of social deprivation, as defined by an index based on different factors such as the presence of universal health insurance, educational level, occupational status, household income or living with a partner, and identified results that were different from those of our study. In that analysis, the adjusted model indicated that their index was associated with late prenatal care (OR 5.8, 95% CI 4.6 to 7.2) and small for gestational age (OR 1.5, 95% CI 1.1 to 1.9) [14].

As previously described, we also found that patients originating from medium/low HDI countries had a higher probability of spontaneous PTD before 37 weeks of gestation relative to their higher HDI counterparts. Some authors have suggested that a lower level of maternal education may be an underlying cause of the increased early delivery risk observed in less educated populations. These patients may be poorer and have other comorbidities, both of which have been identified as independent risk factors for preterm birth. These patients may also be less involved in decision making and planning during pregnancy, and a previous study suggested that a greater proportion of these women believed that delivery at 34 gestational weeks was safe [15, 16]. A population study including more than 500,000 singleton pregnancies in the UK reported Afro-Caribbean patients to be at 1.6 times greater odds of PTD before 37 gestational weeks than Caucasian patients [17].

Other authors using social deprivation variables and other poverty indicators have not identified an association between these factors and preterm birth [18].

The manner in which maternal origin and ethnicity are associated with PE remains poorly understood. Without knowledge of the underlying causes of disease, we can only explore risk factors. A retrospective cohort study including 127,544 low risk women in the USA examined the role of maternal ethnicity in prenatal outcomes using multivariate models adjusted for various confounding variables, and the rates of PE were higher among African American than white women (OR 1.49, CI 95% 1.33–1,72) [19]. Maternal origin may also be associated with hypertensive disorders in pregnancy due to its link with socioeconomic status, which has demonstrated an inverse relationship with PE development.

In our centre, immigrant women had greater odds of poor neonatal outcomes, including abnormal CTG results, CS due to abnormal CTG results and Apgar scores at 5 min. A large study conducted in Canada that included more than 800,000 births showed that the rate of neonatal ICU admissions was higher among infants born to immigrants from South Asia, Africa and the Caribbean than the infants of native Canadian mothers (RR 1.41 CI 95%, 1.36 to 1.46) [20]. Newborns admitted to the ICU may be at increased risk of neonatal mortality and have a significantly higher probability of long-term morbidity [21].

Country of origin has been used to identify maternal and perinatal outcomes; in some regions, the relative risk of neonatal ICU admission has been found to differ between mothers and newborns [22].

### Strengths and limitations

The main strengths of our study are its large sample size and the low rate of missing perinatal outcome data. Our findings regarding the association between maternal origin and pregnancy results are consistent with the results of prior studies.

However, our research also has several limitations. First, we did not consider other maternal and pregnancy characteristics that may be relevant confounders such as maternal smoking, body mass index or method of conception.

Assessments of a mother’s social situation and level of poverty should include educational achievement, maternal and family income, Spanish language acquisition and familial support level. We attempted to divide the patients into groups in a simple manner that was based on maternal HDI; additionally, we obtained as much information as we could regarding the mothers’ social situation, as indicated by their country of origin.

We also lacked information about cultural and health behaviours, other maternal medical conditions such as chronic hypertension and rheumatological diseases, and antenatal care, which precluded us from assessing their impact on pregnancy outcomes. Among immigrants, adverse birth outcomes have been found to be associated with inadequate obstetric care due to difficulties accessing medical services [23].

We used the Strengthening the Reporting of Observational Studies in Epidemiology (STROBE) statement checklist as an instrument to evaluate the quality of our work and assure the presence of items that should be included in reports of observational studies [24].

## Conclusions

In conclusion, this study contributes to the body of literature by generating a better understanding of the manner in which maternal HDI may serve as a score that integrates crucial social factors related to pregnancy and perinatal outcome in our country. We identified lower odds of LBW among immigrants from lower HDI countries despite the identification of greater odds of PTD before 37 gestational weeks, PE and poorer neonatal outcomes in this group.

These findings emphasize the importance of assessing maternal socioeconomic status to facilitate an understanding of the relationship between migration and perinatal outcome. Future studies should continue to evaluate the impact of political and economic interventions on pregnancy outcomes in socially deprived populations.

Further research should also be conducted to more accurately assess the socioeconomic characteristics of immigrant populations and their association with obstetrical outcomes. Generating increased knowledge of these characteristics and their associations requires the use of tools that can provide information regarding adherence to pregnancy follow up, programmes, access to prenatal care, and other indicators linked to the use of medical services.

## Abbreviations

CDC: Centers for Disease Control and Prevention
CS: C-Section
CTG: Cardiotocography
GDM: Gestational diabetes mellitus
GNI: Gross national income per capita
HDI: Human Development Index
ICU: Intensive care unit
MEDC: More economically developed countries
NCHS: National Centre for Health Statistics
PE: Preeclampsia
PTD: Preterm delivery
UN: United Nations
UNDP: United Nations Development Programme
WHO: World Health Organization
